# 11-Deoxycortisol controls hydromineral balance in the most basal osmoregulating vertebrate, sea lamprey (*Petromyzon marinus*)

**DOI:** 10.1038/s41598-020-69061-4

**Published:** 2020-07-22

**Authors:** Ciaran A. Shaughnessy, Andre Barany, Stephen D. McCormick

**Affiliations:** 1 Graduate Program in Organismic and Evolutionary Biology, University of Massachusetts, Amherst, MA USA; 2 Departamento de Biología, Universidad de Cádiz, Cádiz, Spain; 3 Department of Biology, University of Massachusetts, Amherst, MA USA; 4 U.S. Geological Survey, Leetown Science Center, S.O. Conte Anadromous Fish Research Center, Turners Falls, MA USA

**Keywords:** Molecular evolution, Animal physiology, Steroid hormones

## Abstract

It is unknown whether and how osmoregulation is controlled by corticosteroid signaling in the phylogenetically basal vertebrate group Agnatha, including lampreys and hagfishes. It is known that a truncated steroid biosynthetic pathway in lampreys produces two predominant circulating corticosteroids, 11-deoxycortisol (S) and 11-deoxycorticosterone (DOC). Furthermore, lampreys express only a single, ancestral corticosteroid receptor (CR). Whether S and/or DOC interact with the CR to control osmoregulation in lampreys is still unknown. We examined the role of the endogenous corticosteroids in vivo and ex vivo in sea lamprey (*Petromyzon marinus*) during the critical metamorphic period during which sea lamprey increase osmoregulatory capacity and acquire seawater (SW) tolerance. We demonstrate in vivo that increases in circulating [S] and gill CR abundance are associated with increases in osmoregulatory capacity during metamorphosis. We further show that in vivo and ex vivo treatment with S increases activity and expression of gill active ion transporters and improves SW tolerance, and that only S (and not DOC) has regulatory control over active ion transport in the gills. Lastly, we show that the lamprey CR expresses an ancestral, spironolactone-as-agonist structural motif and that spironolactone treatment in vivo increases osmoregulatory capacity. Together, these results demonstrate that S is an osmoregulatory hormone in lamprey and that receptor-mediated discriminative corticosteroid regulation of hydromineral balance is an evolutionarily basal trait among vertebrates.

## Introduction

Corticosteroid signaling is central in controlling many physiological functions in vertebrates including metabolism, ion homeostasis and the stress response. Although the role of corticosteroids in controlling physiological function has been described in most vertebrate groups, the physiological role of corticosteroids and their receptor(s) in the phylogenetically basal vertebrate group Agnatha, represented by extant lamprey and hagfish, is not well understood. Recent investigations indicate that the terminal corticosteroids cortisol (F) and aldosterone (A) are absent in lamprey serum, whereas 11-deoxycortisol (S) and 11-deoxycorticosterone (DOC), respective steroid biosynthetic precursors to F and A in more derived vertebrates, are present at physiologically relevant levels^1–3^. Still, it remains unclear whether the Agnathan corticosteroid receptor (CR), which is understood to be ancestral to the appearance and divergence of the mineralocorticoid (MR) and glucocorticoid (GR) receptors of later vertebrates^4^, has affinity for and is activated by S, DOC or both. For instance, one approach using classical ex vivo receptor binding studies demonstrated that the lamprey CR only had affinity for S^1^, yet a different approach expressing the ligand binding domain of the lamprey CR in mammalian cells in vitro demonstrated the lamprey CR can be activated by several corticosteroids including both endogenous terminal corticosteroids in lamprey, S and DOC^2^. This raises the question of whether S or DOC or both act as a hormone in the basal vertebrate group, and thus, there is a need to better understand the true physiological role of the lamprey CR and its interaction with the endogenous corticosteroids, S and DOC. Physiological approaches in lamprey will address this gap in knowledge and provide a more complete understanding of the endocrine action, particularly the osmoregulatory role, of corticosteroids and their receptors in the basal vertebrates^5^.

Among agnathans, only the lampreys are osmoregulators, whereas all hagfish species are osmoconformers. Sea lamprey (*Petromyzon marinus*) is an ideal model to study the evolution of hormonal control of osmoregulatory processes due to its basal phylogenetic position and its complex and fascinating life history, which includes a migration from freshwater (FW) to seawater (SW) following a larvae-to-juvenile metamorphosis. After 4–6 years of larval life in FW with no ability to survive in SW, sea lamprey undergo metamorphosis during which major morphological and physiological changes occur, including the development of an eye and a toothy, oral disc^6^, proliferation of branchial salt-secreting cells called ‘ionocytes’^7^, upregulation of hypo-osmoregulatory mechanisms in the gill and intestine^8–11^, and acquisition of SW tolerance^8–12^. After metamorphosis, juvenile sea lamprey migrate downstream into the sea to feed parasitically and grow into adulthood.

In fishes, the transition from FW to SW includes a transformation of the gill epithelium from a site of ion uptake to a site of salt secretion. The transcellular secretion of Cl^−^ and paracellular secretion of Na^+^ are known to involve basolateral Na^+^/K^+^-ATPase (NKA) and Na^+^/K^+^/2Cl^–^cotransporter (NKCC1)^13^. Corticosteroid control of salt-secretory mechanisms and SW tolerance in teleost fishes are well-documented^14^. Numerous studies in euryhaline fishes—those which can survive in both FW and SW—have shown that cortisol has an important role in controlling osmoregulatory processes^15^. In anadromous salmonids, for instance, circulating concentrations of cortisol increase during the parr-smolt transformation (which includes development of SW tolerance)^16^, in vivo treatment with cortisol increases gill NKA activity and SW tolerance^17–21^, and the action of cortisol on affecting active ion transport in the gills of salmonids is mediated mainly through the GR rather than the MR^18,22,23^. In teleosts, although DOC signaling acting through the MR has a more minor role in osmoregulation, it has been implicated in partially regulating several osmoregulatory processes, including ion transport and paracellular permeability^14,24^.

Lampreys possess only a basal CR, rather than the diverged GR and MR of later vertebrates^25^. Recentley, discrepant reports from in vitro and ex vivo studies have been published regarding the binding and transactivation of the lamprey CR by the endogenous lamprey corticosteroids, S and DOC. Thus, there is a clear need for more in vivo studies to discern the true physiological role of corticosteroid signaling in controlling hydromineral balance in this basal vertebrate group.

In the present study, we address this conspicuous gap in our current understanding of the role of corticosteroids in Agnatha by using in vivo and ex vivo experimentation to determine the physiological importance and specificity of corticosteroid control of osmoregulation in the sea lamprey. We focus on the critical developmental period of metamorphosis when there are critical changes in osmoregulatory physiology associated with seawater entry. We seek to relate osmoregulatory function to functional properties of the lamprey CR and, specifically, to discern (i) whether circulating S and/or gill CR drive osmoregulatory changes during metamorphosis and (ii) whether S, DOC, or both endogenous corticosteroids control major osmoregulatory changes during sea lamprey metamorphosis. Through this work, our objective was to resolve whether the basal osmoregulating vertebrate, which expresses only a single corticosteroid receptor, has the capacity for discriminative corticosteroid control over physiological processes of hydromineral balance, or if this is an evolved trait among vertebrates which occurred after the MR/GR divergence.

## Results

### Lamprey gill CR has high affinity and specificity for 11-deoxycortisol over other endogenous and later-evolved corticosteroids

**Figure 1:**
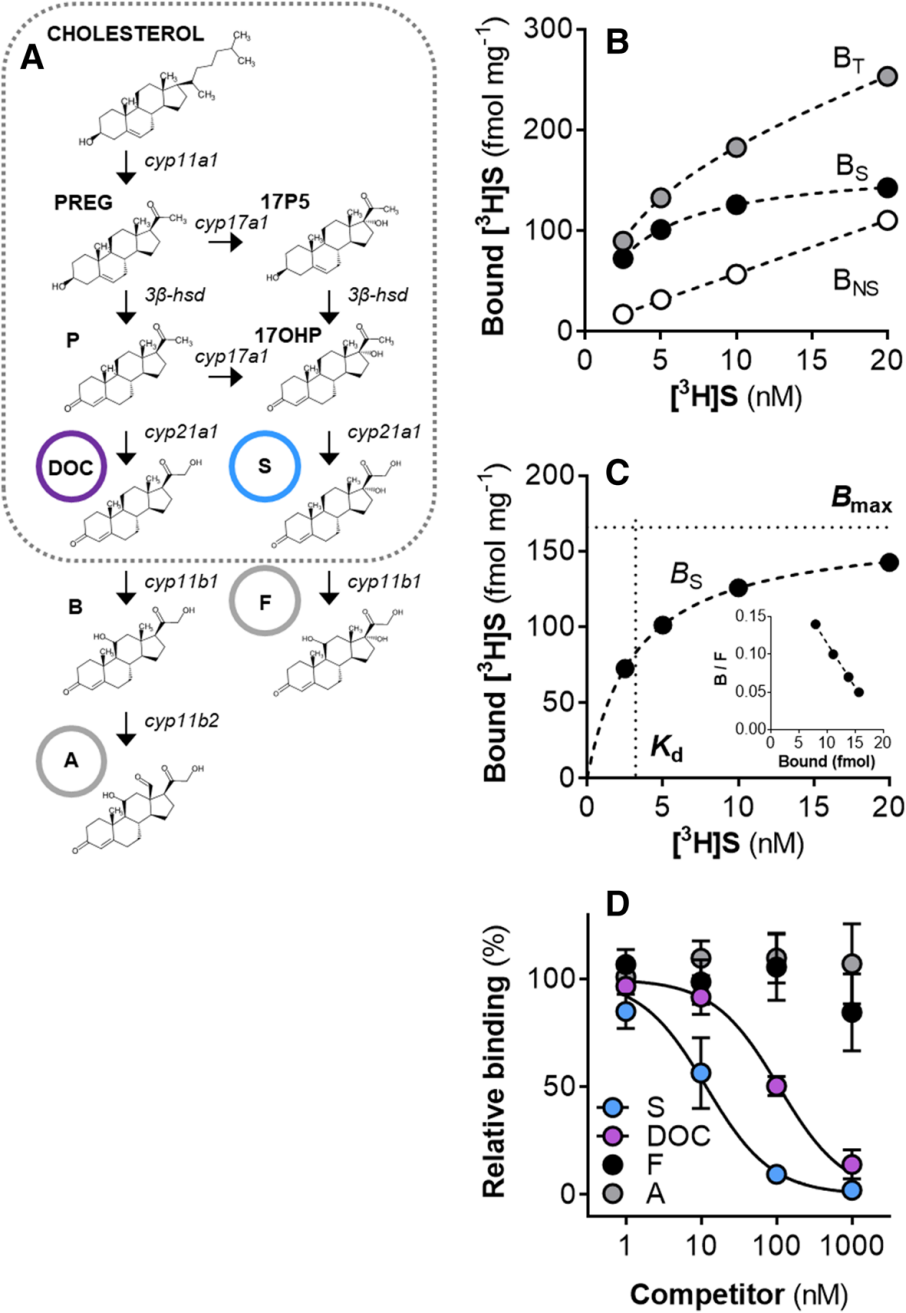
Lamprey corticosteroid biosynthesis and characterization of the lamprey CR. (**A**) Corticosteroid biosynthetic pathway; dotted box represents the pathway components present in lamprey (*cyp11b* genes are absent in lamprey). Corticosteroid abbreviations: PREG, pregnenolone; 17P5, 17-hydroxypregnenolone; P, progesterone; 17OHP, 17-hydroxyprogesterone; DOC, deoxycorticosterone; S, 11-deoxycortisol; B, corticosterone; A, aldosterone. Chemical structures were drawn using MarvinSketch Version 20.13 (ChemAxon; https://www.chemaxon.com). (**B**) Representative receptor binding curves for total binding (B_T_), nonspecific binding (B_NS_), and specific binding (B_s_ = B_T_ − B_NS_). (**C**) Representative specific binding saturation curve showing calculated values for corticosteroid receptor abundance (*B*_max_) and equilibrium dissociation constant (*K*_d_); insert: Rosenthal plot of saturation curve (B = bound, F = free). (**D**) Relative binding affinity for endogenous (S and DOC) and later-evolved (F and A) corticosteroids (*n* = 3; values represent mean ± SEM); IC_50_ (half-maximal inhibition) values: IC_50_(S) = 15.7 nM; IC_50_(DOC) = 89.1 nM (unpaired *t* test: *P* < 0.001; *n* = 3).

The physiological corticosteroids in lamprey, S DOC, are produced from a truncated steroid biosynthesis pathway in lieu of the existence of *cyp11b* genes in the lamprey genome (Fig. 1A). We performed classical receptor binding assays on larval and metamorphic lamprey gill tissue ex vivo to characterize binding properties of the lamprey gill CR. We also assessed binding affinity of the lamprey gill CR to endogenous (S and DOC) and later-evolved (F and A) corticosteroid ligands. Saturable binding by S in the lamprey gills produced curves used to calculate specific binding (*B*_S_), dissociation constant (*K*_d_), and abundance (*B*_max_) for the lamprey gill CR (representative curves presented in Fig. 1B,C). The binding affinity hierarchy of the lamprey gill CR to corticosteroids was S > DOC >  > F = A (Fig. 1D); the lamprey gill CR had significantly greater binding affinity (IC_50_; half-maximal inhibition) for S (15.7 nM) than DOC (89.1 nM), or F and A (no binding detected).

### Osmoregulatory changes in the lamprey gill during metamorphosis are related to circulating 11-deoxycortisol and gill CR abundance

**Figure 2:**
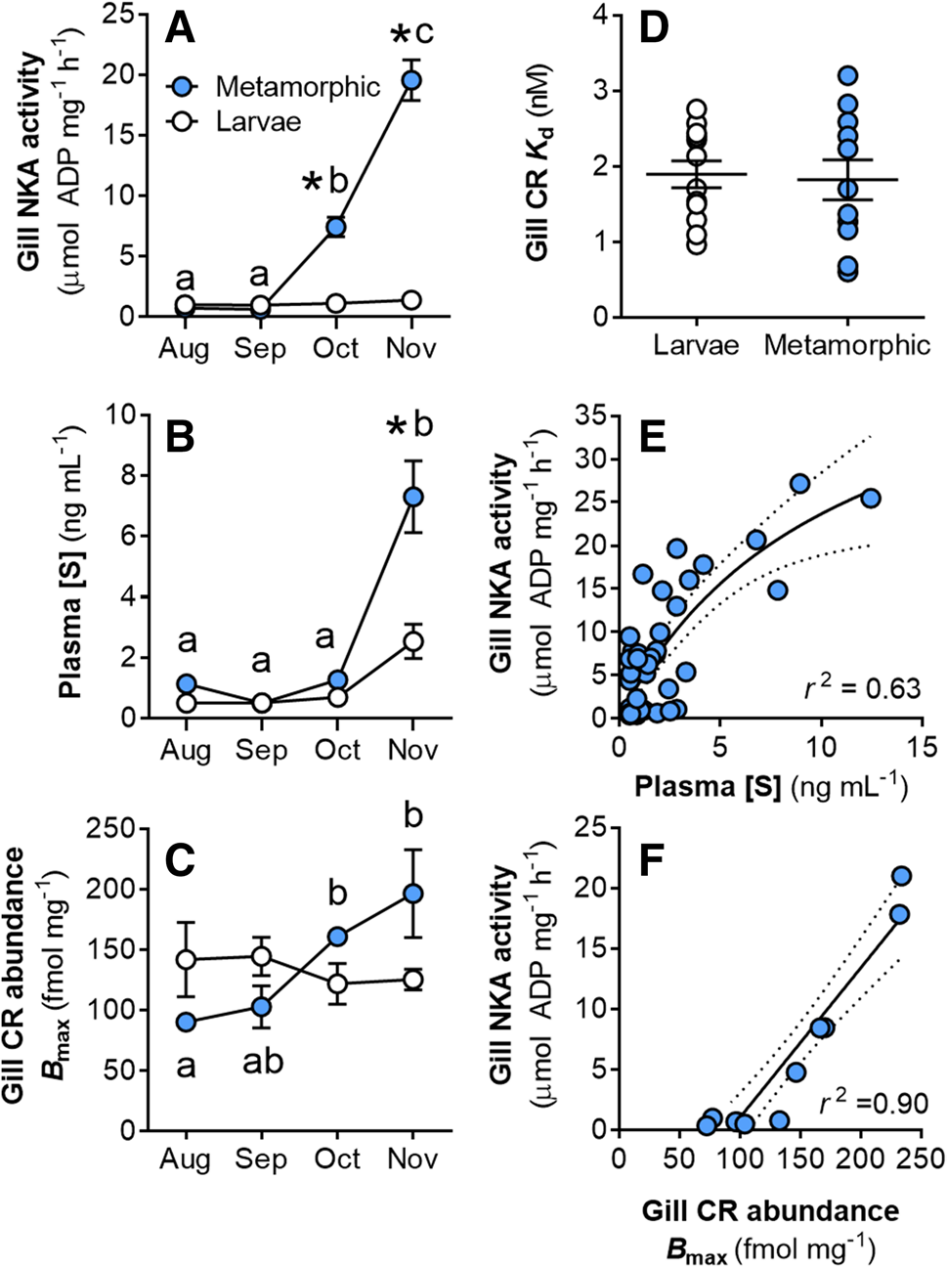
Osmoregulatory and endocrine changes during sea lamprey metamorphosis. (**A**–**C**) Changes in gill NKA activity (*n* = 8–12; *P*_LS,MO,INT_ < 0.001), plasma 11-deoxycortisol concentration ([S]) (*n* = 8–12; *P*_LS,MO,INT_ < 0.01), and gill CR abundance (*B*_max_) (*n* = 3; *P*_INT_ < 0.01) during sea lamprey metamorphosis. Values represent mean ± SEM, letters indicate differences among metamorphic lamprey (blue), and asterisks indicate differences from time-matched larvae (white) (two-way ANOVA; Tukey’s post hoc; LS, life stage; MO, month; INT, interaction). (**D**) Dissociation constant (*K*_d_) from larvae and metamorphic individuals (unpaired *t* test: *P* = 0.817; *n* = 12). Horizontal lines indicate mean ± SEM. (**E**,**F**) Relationships between gill NKA activity and plasma [S] (hyperbolic) and gill CR abundance (*B*_max_) (linear; *P* < 0.001) in metamorphic sea lamprey. Dotted lines represent 95% confidence bands.

We compared gill NKA activity (a widely used metric of osmoregulatory capacity in fishes), circulating [S], and gill CR expression between larval and metamorphic lamprey in vivo, and assessed the relationships of gill NKA activity with gill CR abundance and plasma [S]. Gill NKA activity in early- and mid-metamorphic lamprey (Aug–Sep) remained near larval levels at 1–2 μmol ADP mg^−1^ h^−1^ and increased to 19.5 ± 1.7 μmol ADP mg^−1^ h^−1^ by the final stages and completion of metamorphosis (Nov) (Fig. 2A). Plasma [S] increased above larval levels of ~ 1 to a maximum of  7.3 ± 1.1 ng mL^−1^ in the final month of metamorphosis (Fig. 2B). Gill CR abundance in larvae remained steady at ~ 109–155 fmol mg^−1^ (interquartile range), but gill CR abundance in metamorphosing lamprey increased over threefold, from 90 ± 6 in Aug to 196 ± 36 fmol mg^−1^ in Nov (Fig. 2C). The dissociation constant of the gill CR was not different between larval and metamorphic lamprey (*K*_d_ = 1.2–2.6 nM, interquartile range) (Fig. 2D). The relationship between gill NKA activity and plasma [S] during metamorphosis was hyperbolic (*y*_max_ = 48.8 ± 15.8 μmol ADP mg^−1^ h^−1^; *y*_50%_ = 10.7 ± 5.1 ng mL^−1^; *r*^2^ = 0.63; Fig. 2E). The relationship between gill NKA activity and gill CR abundance during metamorphosis was linear (*P* < 0.001; *F*_1,10_ = 84.8; *m* = 0.12 ± 0.01; *r*^2^ = 0.90; Fig. 2F).

### 11-Deoxycortisol treatment improves osmoregulatory capacity in metamorphosing lamprey

**Figure 3:**
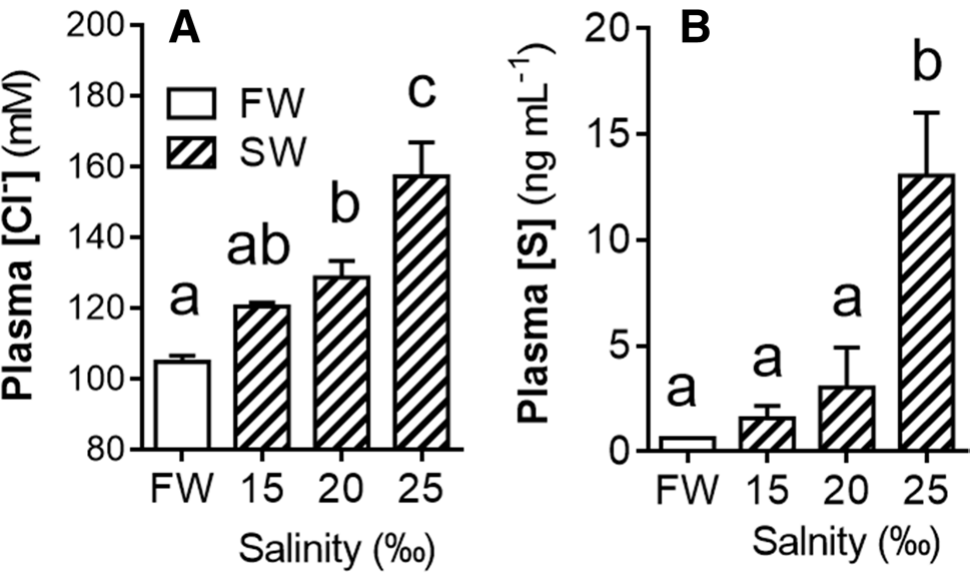
Plasma ion and corticosteroid response to SW exposure. Changes in concentrations of (**A**) plasma chloride ([Cl^−^]) (*P* < 0.001) and (**B**) plasma 11-deoxycortisol ([S]) (*P* = 0.007) in mid-metamorphic (early-Oct) sea lamprey after 24 h exposure to freshwater (FW) or various dilutions of seawater (SW; dashed bars). Values represent mean ± SEM and data points with different letters are significantly different (*n* = 4–6; one-way ANOVA; Tukey’s post hoc).

**Figure 4:**
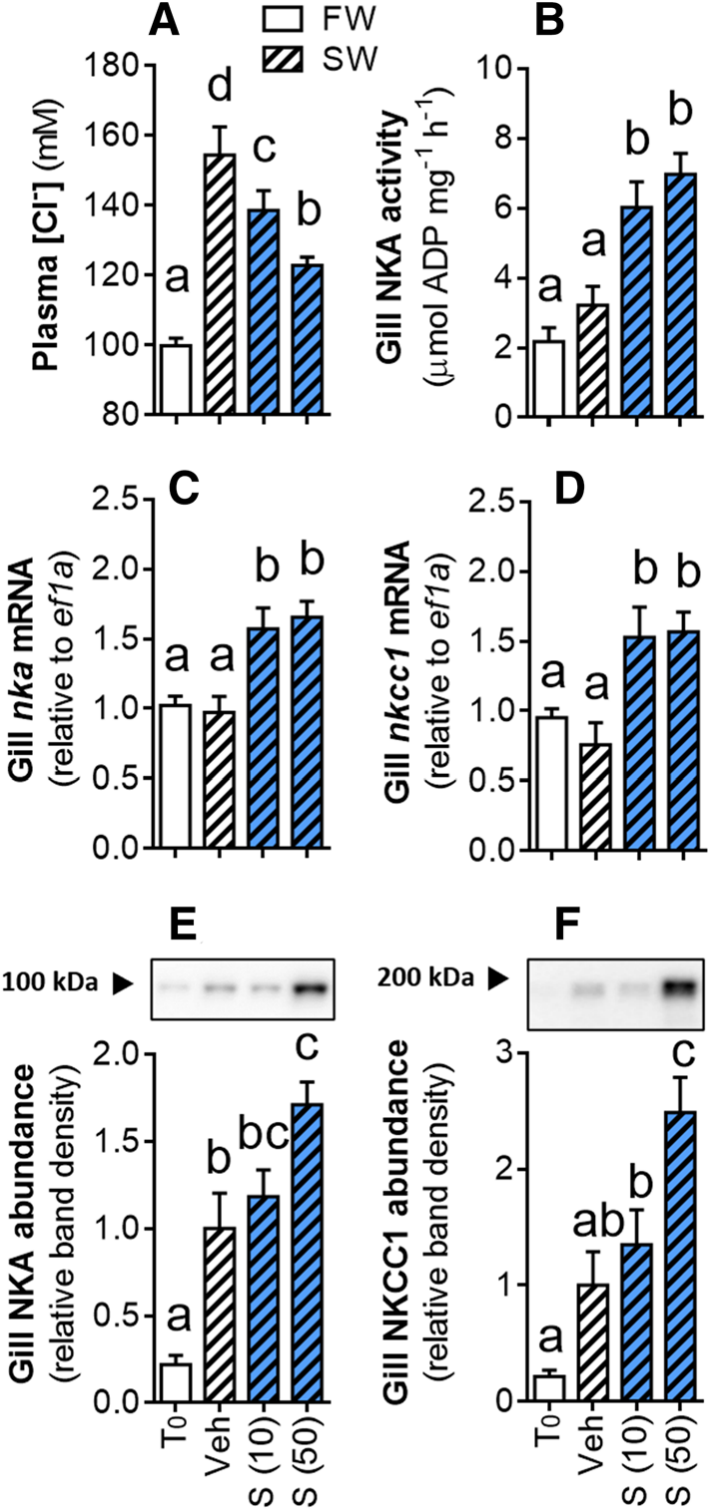
Effects of in vivo S treatment on lamprey osmoregulation after SW exposure. Mid-metamorphic sea lamprey were administered S or a vehicle control (Veh), then exposed 12 days post-injection to 25‰ SW for 24 h. (**A**) Plasma [Cl^−^], (**B**) Gill NKA activity. (**C**,**D**) gill mRNA abundance of *nka* and *nkcc1*. (**E**,**F**) Representative bands and quantification of gill NKA and NKCC1 protein abundance. T_0_ = uninjected FW control sampled at time of S administration. Dose of S is stated in parenthesis (μg g^−1^ body weight). Values represent mean ± SEM and data points with different letters are significantly different (*n* = 8–10; one-way ANOVA (**A**–**F**: *P* < 0.001); Tukey’s post hoc).

**Figure 5:**
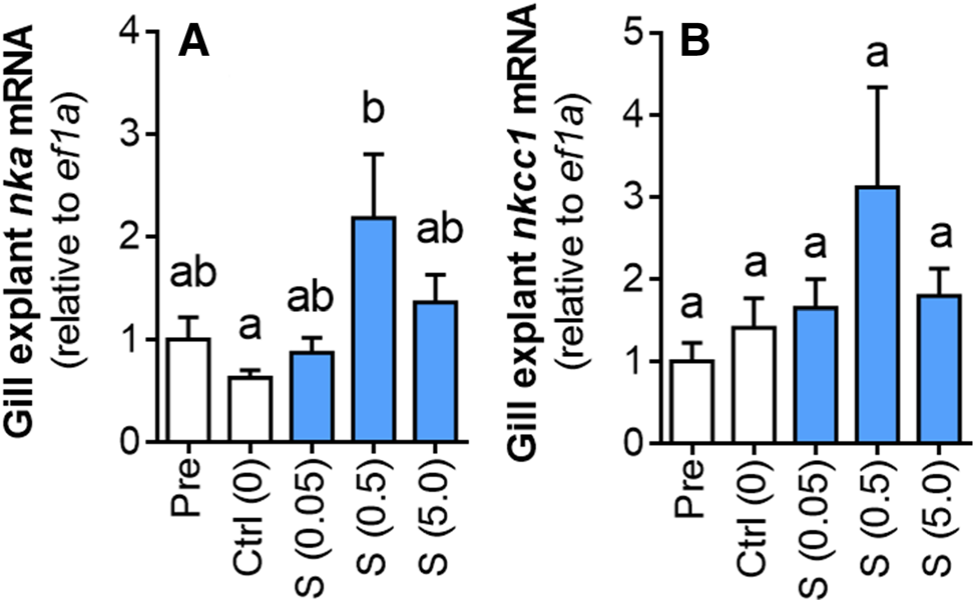
Effects of ex vivo S treatment on gill ion transporter expression. Changes in abundance of (**A**) *nka* mRNA (*P* < 0.05) and (**B**) *nkcc1* mRNA (*P* = 0.24) in gill explants taken from mid-metamorphic sea lamprey incubated ex vivo with varying concentrations of S for 24 h. Dose of S is stated in parenthesis (μg mL^−1^). Pre = gill tissue prior to incubation; Ctrl = media only. Values represent mean ± SEM and data points with different letters are significantly different (*n* = 6; one-way ANOVA; Tukey’s post hoc).

In mid-metamorphic (Oct) lamprey, after the significant increase in gill CR but before the endogenous rise in circulating S, maximal gill NKA activity, and acquisition of SW tolerance, we assessed salinity tolerance and evaluated whether treatment with S in vivo (12 days with intraperitoneal S implant) and ex vivo (explanted gill tissue incubated in S for 24 h) would improve osmoregulatory capacity and/or increase expression of key SW gill ion transporters, NKA and NKCC1. We also performed a similar in vivo experiment with larval lamprey, which have very low osmoregulatory capacity and no SW tolerance. Plasma [Cl^−^] and [S] in FW control lamprey (transferred from FW to FW for 24 h) remained at baseline levels of ~ 100 mM and ~ 1 ng mL^−1^, respectively (Fig. 3A,B). Exposure of mid-metamorphic lamprey to elevated salinities for 24 h resulted in salinity-dependent increases in plasma [Cl^−^] and plasma [S]. After 24 h exposure to the highest salinity tested (25‰), plasma [Cl^−^] had risen to ~ 160 mM (Fig. 3A) and plasma [S] to ~ 13 ng mL^−1^ (Fig. 3B). Similar increases in plasma [Cl^−^] (to ~ 155 mM) were observed in the Veh controls transferred to 25‰ (hereafter referred to as “SW”) (Fig. 4A). After 24 h exposure to SW, plasma [Cl^−^] in lamprey treated with 10 and 50 μg g^−1^ S were both maintained closer to FW control levels (~ 140 and ~ 120 mM, respectively) and significantly lower than in the Veh control (Fig. 4A). Lamprey administered S had significantly higher (twofold) gill NKA activity than the Veh control (Fig. 4B), as well as significantly higher gill *nka* (Fig. 4C) and *nkcc1* (Fig. 4D) mRNA abundance. Lamprey administered 50 μg g^−1^ S had increased gill NKA (1.5-fold; Fig. 4E) and NKCC1 (2.5-fold; Fig. 4F) protein abundance over the Veh control. Explanted gill tissue from mid-metamorphic lamprey incubated with S for 24 h ex vivo exhibited a dose-dependent increase in *nka* mRNA expression (Fig. 5A) and a similar trend for *nkcc1* mRNA expression (Fig. 5B). Larval lamprey treated with either dose of S showed no significant increase in gill NKA activity (*P* = 0.297; one-way ANOVA) over the Veh control (Fig. S1). After 24 h exposure to 12‰, larvae plasma [Cl^−^] was elevated from ~ 85 to 110 mM (*P* < 0.001) but there was no significant osmoregulatory improvement from prior treatment with S (*P* = 0.262).

### 11-Deoxycortisol controls osmoregulation in lamprey

**Figure 6:**
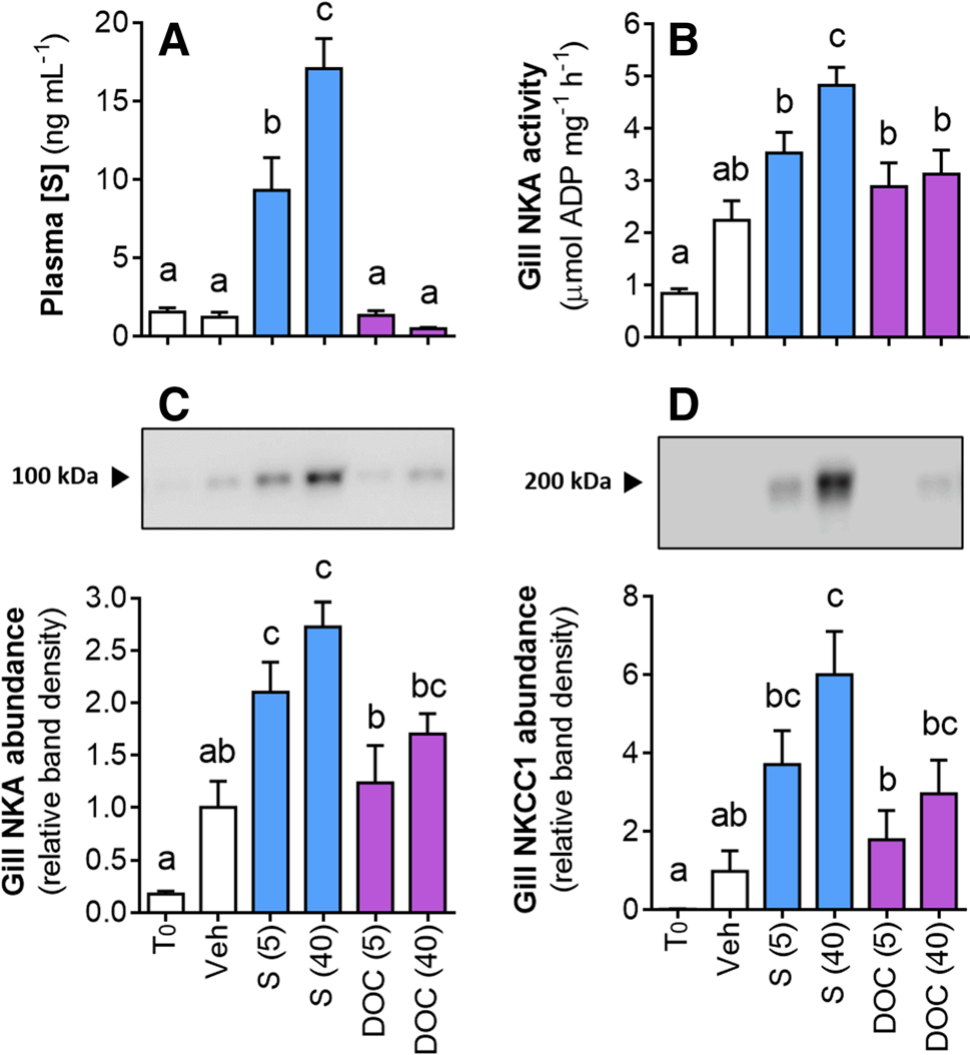
Comparison of the action of endogenous corticosteroids on lamprey osmoregulation in vivo. Mid-metamorphic sea lamprey were administered S, DOC, or a Veh control, then sampled 12 days post-injection. (**A**) Plasma [S]. (**B**) Gill NKA activity. (**C**,**D**) Representative bands and quantification of gill NKA and NKCC1 protein abundance. T_0_ = uninjected control sampled at time of corticosteroid administration. Dose of S and DOC is stated in parenthesis (μg g^−1^ body weight). Values represent mean ± SEM and data points with different letters are significantly different (*n* = 8–12; one-way ANOVA (**A**–**D**: *P* < 0.001); Tukey’s post hoc).

In a similar in vivo experiment using intraperitoneal administration of corticosteroids in metamorphic lamprey, we sought to test whether the endogenous corticosteroids in lamprey, S and DOC, controlled osmoregulation. As expected, intraperitoneal administration of S resulted in a dose-dependent elevation of circulating [S] (Fig. 6A). Compared to the Veh control (~ 2 μmol ADP mg^−1^ h^−1^), gill NKA activity was significantly elevated by treatment with S, but treatment with DOC did not elevate gill NKA activity (Fig. 6B). Similarly, gill NKA and NKCC1 protein abundance were significantly elevated (~ threefold and ~ sixfold, respectively) by treatment with S but neither were elevated by treatment with DOC (Fig. 6C,D).

### Ancestral MR-like structural properties of the lamprey CR confer osmoregulatory control by spironolactone

**Figure 7:**
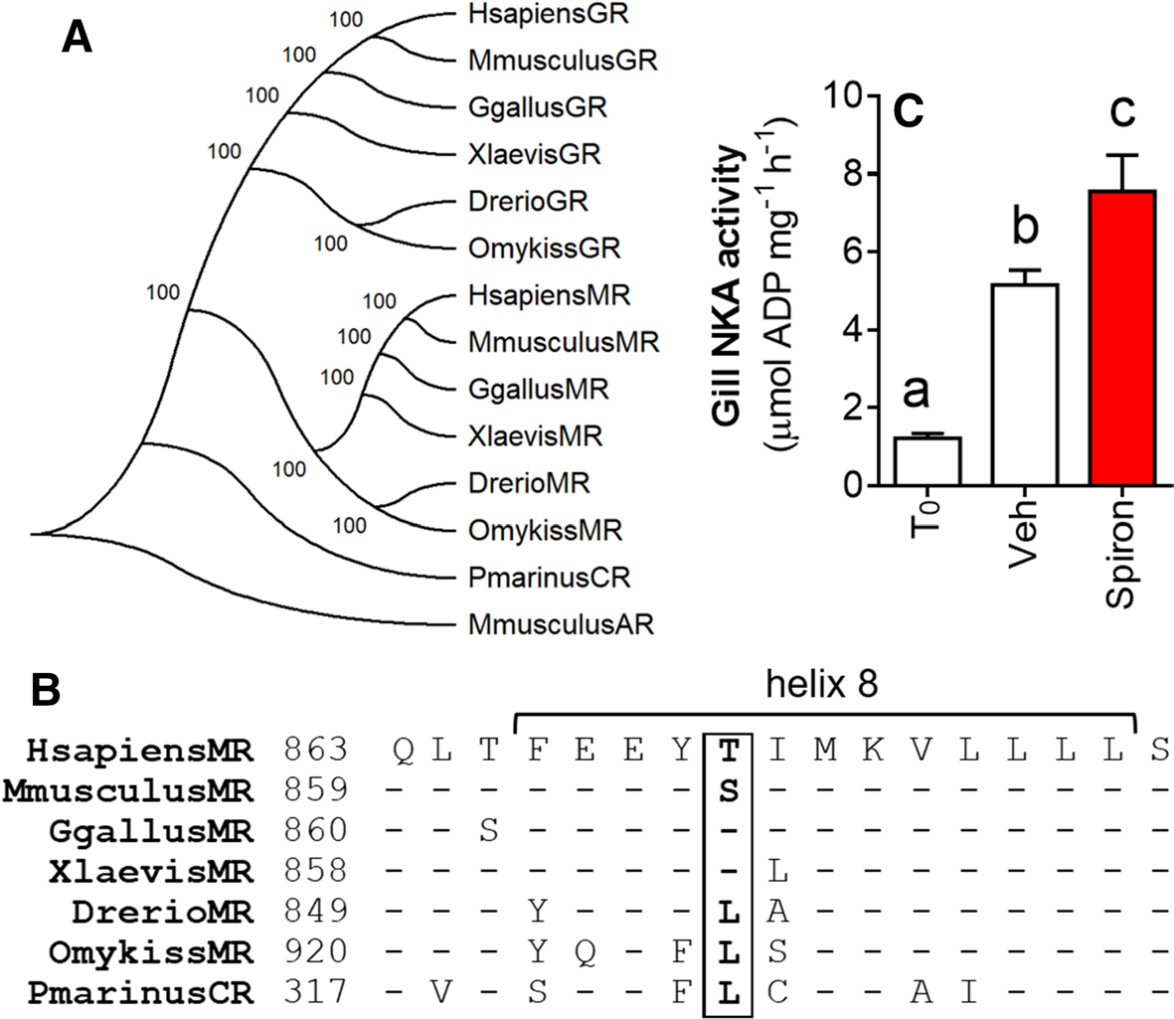
Molecular phylogeny of vertebrate corticosteroid receptors and mineralocorticoid-like action of spironolactone in lamprey. (**A**) Lamprey CR placed among a phylogeny of vertebrate MR and GR coding sequences (rooted on mouse androgen receptor, AR). Molecular phylogenetic analysis using peptide sequence data obtained from NCBI Genbank were performed using ClustalW alignment (https://www.ebi.ac.uk/clustalw) implemented by MEGA7 software (https://www.megasoftware.net). (**B**) Sequence alignment of helix 8 of the vertebrate MR and lamprey CR. Box indicates the key evolutionary substitution of ancestral Leu with Thr (Ser in rodents) that conferred antagonistic action of spironolactone in tetrapods. (**C**) Gill NKA activity in mid-metamorphic sea lamprey administered spironolactone (200 μg g^−1^ body weight) or a Veh control, then sampled 21 days post injection. T_0_ = uninjected control sampled at time of spironolactone administration. Values represent mean ± SEM and data points with different letters are significantly different (*n* = 8–12; one-way ANOVA; *P* < 0.001; Tukey’s post hoc).

In a final experiment, we sought to relate structural properties of the lamprey CR to osmoregulatory function*.* It was recently determined that a key Leu-to-Thr (Ser in rodents) substitution in helix 8 of the vertebrate MR is critical for switching the action of spironolactone from an agonist to an antagonist. In a phylogeny of vertebrate corticosteroid receptors, the lamprey CR occupies a position that precedes the divergence of MR and GR (Fig. 7A). The lamprey CR possess the ancestral Leu at residue 810 (on human MR) on helix 8 (Fig. 7B), suggesting that spironolactone should be an agonist of the lamprey CR. Consistent with this prediction, in vivo treatment with spironolactone increased gill NKA activity (Fig. 7C).

## Discussion

As the most phylogenetically basal vertebrates with an osmoregulatory strategy, lampreys are the ideal model organism to gain insight into the appearance and evolution of endocrine control of hydromineral balance in vertebrates. This work provides several novel lines of evidence that S is the primary corticosteroid hormone that acts through the ancestral CR to control osmoregulation in the basal vertebrate, sea lamprey: (i) plasma [S] and gill CR abundance are upregulated during metamorphosis when endogenous increases in salinity tolerance occur; (ii) plasma [S] is upregulated during SW exposure; (iii) ex vivo and in vivo treatment with S upregulates expression of critical active gill ion transporters and increases SW tolerance; (iv) in vivo treatment with the other known circulating corticosteroid in lamprey, DOC, does not upregulate such transcellular ionoregulatory machinery; and (v) treatment with a pharmacological agonist of the lamprey CR, spironolactone, is effective in stimulating lamprey gill ion transporter activity similar to the effects of S.

To understand how corticosteroids control osmoregulation in lamprey, it is necessary to observe the nature of endogenous osmoregulatory and endocrine changes that occur naturally during metamorphosis and SW acclimation. In the present study, we show large increases in gill NKA activity during the later stages of metamorphosis (Oct and Nov) occurred along with increases in plasma [S] and gill CR abundance. The increase in gill NKA activity during metamorphosis corresponds with what has been observed previously with gill NKA (activity and protein abundance^8–10^) as well as gill NKCC1 protein abundance^9^. An increase in gill CR abundance was detected earlier in metamorphosis (Oct) compared to that of plasma [S] (Nov). It makes intuitive sense that an increase in the intrinsic capacity of the lamprey gill to receive a corticosteroid signal (i.e., increase in gill CR abundance) would occur prior to an elevation of the concentration of circulating corticosteroid, whereas the opposite case (upregulating plasma [S] before increasing gill CR abundance) would less efficiently utilize the circulating hormone. A similar endocrine program is present in anadromous salmonids in which, along with circulating cortisol, GR abundance (as measured by binding studies similar to those used in the present study) is upregulated during the parr-smolt transformation ahead of SW entry^26–30^. In teleost fishes, the GR is known to be important in mediating osmoregulatory adaptation^23,31^. The hyperbolic relationship between lamprey gill NKA and plasma [S] shown in the present study suggests a saturating potential of the hormone—at some concentration of circulating S, the CRs in the gill become saturated and a further increase in plasma [S] does not further stimulate gill NKA activity. Indeed, the apparent saturating effect of [S] on gill NKA activity occurs around ~ 5–10 ng mL^−1^ (NKA activity at 50% = 10.7 ± 5.1 ng mL^−1^), or ~ 15–30 nM, which closely approximates the range of calculated values for IC_50_ of the gill CR to S of ~ 15 nM. Conversely, gill NKA activity was more directly related to increases in gill CR abundance, suggesting that the receptor is important in directly mediating the corticosteroid signal and affecting an osmoregulatory response in the metamorphic sea lamprey. In anadromous salmonids, increases in GR abundance during the parr-smolt transformation is regulated by growth hormone (GH)^32^. GH has been identified in sea lamprey^33^, but more work is needed to determine whether osmoregulation is controlled by a similar endocrine program in sea lamprey.

Mid-metamorphic (early-Oct) lamprey exposed to elevated salinity elicit a salinity-dependent increase in both plasma [Cl^−^] and [S] (Fig. 3), whereas it is known that fully metamorphosed lamprey exhibit little to no loss of ion homeostasis after SW exposure^10^. The increase in plasma [S] upon SW exposure indicates that SW exposure early in metamorphosis (before hypo-osmoregulatory preparation is complete) is either stressful and/or that circulating [S] increases to promote salt secretion. In either case, S signaling seems important for lamprey to initiate osmoregulatory changes necessary to survive in SW when osmoregulatory capacity is low.

Ex vivo and in vivo experimentation with cortisol have proven a useful method for establishing its role in osmoregulatory control in other vertebrates^14^, and so we used similar approaches to characterize the osmoregulatory role of S in sea lamprey. Our hormone treatment and salinity experiments were carried out in October, which we had determined to be the best time to conduct these experiments as it was after the significant increase in gill CR abundance, but prior to the endogenous increases in plasma [S] and peak gill NKA activity. During this time frame, SW tolerance is higher than in larvae (which can only tolerate a maximum of 12‰) but still lower than fully transformed juveniles^9^. After 24 h in elevated salinity, metamorphosing lamprey in October experienced salinity-dependent loss of osmotic homeostasis, evidenced by salinity-dependent increases in plasma [Cl^−^] to well above ~ 120 mM [Cl^−^] (Figs. 3, 4), which is the maximum ion disturbance experienced by fully metamorphosed juveniles^8^. Prior treatment with S clearly improved osmoregulatory capacity as evidenced by an enhanced ability to maintain lower levels of plasma [Cl^−^] after SW exposure compared to Veh-injected controls. Differences observed between T_0_ and Veh in gill ion transporter activity and expression reflects the upregulation of these osmoregulatory machinery during the natural progression of metamorphosis^8,9^ and is not indicative of an artifactual effect of the Veh. The effect of S treatment on plasma [Cl^−^] after SW transfer was dose-dependent. If S signaling for gill osmoregulatory changes is indeed CR-mediated, this dose-dependency indicates that gill CR is present at levels sufficient to respond to a range of circulating S concentrations. The enhanced osmoregulatory ability in lamprey treated with S is likely due to the corresponding increases in gill ion transporters (NKA and NKCC1) observed, since these transporters also increase during metamorphosis when salinity tolerance increases. Similar increases in gill NKA and NKCC1 have also been observed in conjunction with higher levels of salinity tolerance in teleost fishes treated with cortisol^15^.

In addition to characterizing the role of S and CR during metamorphosis, we sought to investigate the specificity of corticosteroid action in vivo by conducting a similar experiment administering both endogenous corticosteroids, S or DOC. We confirmed that our method of intraperitoneal injection with S achieved dose-wise increases in circulating [S] within a physiological range, and replicated the results from Fig. 4 showing an increase in gill NKA and NKCC1 occurs after treatment with S. In contrast to the stimulating effects of S, we observed that the other endogenous corticosteroid, DOC, did not significantly upregulate such transcellular active gill ionoregulatory machinery. The lack of effect of DOC on active gill ion transport is likely due to the relatively low binding affinity for DOC compared to S by the lamprey gill CR, as first reported by Close et al.^1^ and replicated in the present study. It is also possible that in situ inactivation of DOC by a reductase (e.g., 5α-reductase) or some other pathway of steroid catabolism is occurring in the gill, much like how hydroxysteroid dehydrogenases (HSDs) are known to regulate tissue-specific physiologic actions to corticosteroids in other vertebrates^34^.

It must be acknowledged that, in addition to its role in transcellular active ion transport demonstrated in the present study, S may also have a role in lamprey osmoregulation through other mechanisms such as control of paracellular ion and water transport. It is known that tight junction (TJ) complexes dynamically control paracellular permeability in many vertebrate epithelia, including changes in gill epithelium of teleost fishes from “tight” in FW to “leaky” in SW^35^. A functional role for TJ complex proteins has more recently been elucidated in the sea lamprey^35–38^. Several studies have demonstrated putative corticosteroid control of TJ proteins in cultured teleost fish gill epithelia^24,39,40^, and it is possible that S may thus also control TJ complexing and paracellular permeability in the sea lamprey gill, in addition to its role in controlling active ion transport.

Interestingly, we were unable to stimulate an osmoregulatory response in larval lamprey with an identical in vivo approach of S treatment, despite larvae having apparently adequate gill CR abundance (*B*_max_ ~ 100–150 fmol mg^−1^) to bind the administered S. It could be that cell type-specific expression of the lamprey CR is modulating differences in osmoregulatory action of S between larvae and metamorphic lamprey—that the CR is expressed in Cl^−^ secreting gill ionocytes in metamorphic lamprey but not in larval lamprey. Immunological studies using antibodies raised against the lamprey CR are needed to better understand the physiological role and localization of the lamprey CR. Another explanation may be that signaling from a GH/insulin-like growth factor (IGF) pathway is required for initial ionocyte differentiation and upregulation of CR early in lamprey metamorphosis, after which the corticosteroid can then act to upregulate protein abundance of ion transporters, as has been a suggested model of coordinated endocrine control of osmoregulation in teleosts^41^. In any case, the role of S in controlling osmoregulation in metamorphic lamprey is clearly apparent—the correspondence of plasma [S] and calculated gill CR *K*_d_ and IC_50_ values, the concordant rise in systemic S and gill CR, and the specific S effects on the gill ionoregulatory apparatus combine to suggest S is an important hormone contributing to development of SW tolerance in the basal sea lamprey.

Lastly, we sought to obtain further in vivo evidence demonstrating that the CR is important in mediating the osmoregulatory control of S during lamprey metamorphosis. In mammalian research, antagonists of MR (such as spironolactone and eplerenone, derivatives of progesterone) or GR (such as RU486) have been used for more than half a century, due to their anti-aldosterone, -cortisol, and -progesterone activities^42,43^. In fishes, MR and GR antagonists have been used to examine the osmoregulatory roles of the MR- and GR-mediated action of corticosteroids, namely cortisol, with mixed results^18,27,31,43–48^. Recently it has been shown that spironolactone serves as an agonist, not an antagonist, to the MR in basal lineages of fishes^49,50^. It was discovered that a substitution of Leu by Thr (Ser in rodents) on helix 8 of the MR of tetrapods confers the switch of progesterone and its derivative, spironolactone, from MR agonists to antagonists, and it has been suggested that this molecular switch may be causally related to the appearance of aldosterone synthesis and mineralocorticoid function of aldosterone in tetrapods^51^. Analysis of the lamprey CR revealed the key Leu residue was present on helix 8, and thus we should expect that spironolactone would act as a CR agonist. If the osmoregulatory effects of S treatment were indeed acting through the lamprey CR, we would expect to see a similar effect of spironolactone. We observed this expected rise in gill NKA activity after 3 weeks of in vivo treatment with spironolactone (Fig. 6), similar in magnitude to our in vivo experiments with S (Fig. 4). These results suggest an MR-like functional quality of the lamprey CR, which has been previously suggested based on molecular and structural analyses of the lamprey CR^2,25,52,53^. To our knowledge, our in vivo results demonstrating the stimulatory action of progesterone derivatives on osmoregulation are the only such in vivo evidence in any vertebrate lineage predating the actinopterygian-sarcopterygian split.

In conclusion, the present study provides much needed in vivo evidence and physiological context to earlier investigations and conflicting reports regarding corticosteroid function in the basal Agnathans. Our work underscores the importance of in vivo and ex vivo studies and inspires more research into corticosteroid function in Agnathan physiology. We demonstrate that a receptor-mediated signal from S, and not DOC, is a major endocrine process controlling osmoregulatory processes in the sea lamprey. That lamprey exhibit discriminative corticosteroid control of osmoregulation (i.e., stimulation active ion transport processes by S but not DOC) indicates that such corticosteroid function appeared early in vertebrate evolution, perhaps in association with the appearance of an osmoregulatory strategy, which has been maintained in nearly all later-evolved vertebrate lineages.

## Materials and methods

### Experimental subjects and animal care

All animal care and experimentation were carried out using protocols approved by the Internal Animal Care and Use Committee at the University of Massachusetts and the U.S. Geological Survey (Protocol number: 2016-0009) and in accordance with all relevant guidelines and regulation (Guide for Care and Use of Laboratory Animals, National Research Council, Washington, DC). Wild larval and pre-metamorphic lamprey were caught in July by electrofishing on the Sawmill River, a tributary to the Connecticut River in Western Massachusetts, USA. After capture, animals were held in 1.5 m diameter flow-through tanks supplied with Connecticut River water with 10 cm of sand. For the metamorphic profile, larval and metamorphosing lamprey were randomly selected for sampling each month from August to December. For hormone treatment experiments, mid-metamorphic lamprey were held in aquaria (80 L) containing dechlorinated, filtered, and aerated recirculating municipal fresh water kept at 15 °C. Artificial SW dilutions were made using a commercial sea salt mix (Crystal Sea Salt, USA).

### In vivo and ex vivo experimentation

Salinity tolerance testing and in vivo hormone treatments were performed in early October on mid-metamorphic (early stage 7)^6^. The biometric data of the lamprey in these experiments were: length = 14.7 ± 0.3 cm; body mass = 5.4 ± 0.4 g; sex ratio = 50% male, 50% female. In salinity tolerance tests, lamprey were exposed to varying salinities (15, 20, or 25‰) or a FW control and sampled for blood after 24 h. For hormone treatments, lamprey were anesthetized with MS-222 (100 mg L^−1^ buffered by NaHCO_3_, pH 7.4) and injected intraperitoneally with vehicle alone (Veh; 1:1, oil:shortening) or Veh containing S, DOC, or spironolactone, then held in freshwater for 12 days (21 days for spironolactone) prior to sampling in FW or after a 24 h exposure to 25‰ (referred to as “SW”). To account for changes which may be occurring naturally during metamorphosis, uninjected lamprey were sampled on the day of injections as a time = 0 control (T_0_). The doses for S injection were adjusted slightly between experiments (Figs. 4 and 6) to better capture any potential dose-dependent effects of S on gill NKA activity. Lamprey were sampled 21 days after spironolactone injection was to allow additional metamorphic progress to occur between injection and sampling in order to better observe the possibility of spironolactone acting as an antagonist of the lamprey CR and slowing osmoregulatory changes during metamorphosis.

For ex vivo hormone treatment, intact whole gill pouches were excised from FW-acclimated mid-metamorphic lamprey and carefully dissected to unfold them from their basket-like arrangement and cleanly remove the afferent and efferent ends to expose the internal filamental vasculature to incubation media. The final ex vivo branchial unit was 4–8 gill filaments (~ 1 mg of wet tissue). Groups of 6–10 intact filament pieces were placed in incubation media (DMEM containing 5 mM glucose and 100 U mL^−1^ penstrep) containing dissolved hormone such that gill tissue from a single fish would be exposed to every hormone treatment (0.05–5.0 μg mL^−1^ S) and a media-only control (Ctrl). Doses of S for ex vivo culture were chosen according to doses of cortisol used in similar ex vivo organ culture experiments in teleosts^54^. Excised gill tissue was frozen and stored at − 80 °C before incubation (Pre) and after 24 h in culture at 15 °C. Ex vivo gill cultures after 24 h incubation were considered viable if they presented (i) no loss of color, (ii) no excessive mucus accumulation, and (iii) no decrease in housekeeping gene mRNA expression.

### Tissue sampling and analysis

Lamprey were euthanized in MS-222 (200 mg L^−1^ buffered by NaHCO_3_, pH 7.4). Blood was collected via caudal transection into heparinized glass hematocrit tubes and plasma was separated following centrifugation. Gills were dissected and frozen at − 80 °C. Gill tissue for enzyme analyses were placed in SEI buffer (150 mM sucrose, 10 mM EDTA, 50 mM imidazole, pH 7.3) before freezing. Plasma [Cl^−^] was measured using a digital chloridometer (Haake Buchler Instruments Inc., USA).

### Na^+^/K^+^-ATPase activity assay

NKA activity was determined by measuring the ouabain-sensitive ADP production of gill homogenates^55^. Gill tissue was thawed, homogenized in SEID buffer (SEI with 0.1% deoxycholate) and centrifuged at 3,000*g* for 5 min. The resulting supernatant was used in an enzyme-linked kinetic assay, which couples ADP production to NADH reduction in a 1:1 ratio to determine ATPase activity. Protein concentration was determined spectrophotometrically using a bovine serum albumin (BSA) standard curve (BCA Protein Assay, Pierce, USA) and the ouabain-sensitive ATPase activity expressed as μmol ADP mg protein^−1^ h^−1^.

### Radioimmunoassay for analysis of plasma [S]

Plasma [S] was measured using a competitive radioimmunoassay (RIA)^1^. The RIA was carried out in glass culture tubes (10 × 75 mm) using a commercial antibody (Ab; CET-M8, Absolute Antibodies Inc., Redcar and Cleveland, UK; RRID: CVCL_J281) and commercial ^3^H-labeled 11-deoxycortisol ([^3^H]S; American Radiolabeled Chemicals, Inc., St. Louis, MO). Each reaction consisted of 10 μL of plasma or standard sample, 100 μL of PBS assay buffer (50 mM NaH_2_PO_4_, 137 mM NaCl, 0.4 mM EDTA, BSA 0.2% w/v, pH 7.4) containing 5,000 cpm [^3^H]S, and 50 μL of Ab diluted 1:5,000 in assay buffer (determined to be appropriate for 50% [^3^H]S binding). The reactions were prepared on ice, incubated for 1 h at 37 °C, then incubated at 4 °C overnight. After overnight incubation, 500 μL of dextran-coated charcoal (PBS, 0.25% w/v dextran, 2.5% w/v activated charcoal) was added to each reaction and incubated on ice for 15 min. Unbound 11-deoxycortisol that associates with charcoal was pulled out of solution by centrifugation at 2,000*g* for 15 min. A 325 μL aliquot of the supernatant containing S and [^3^H]S bound to Ab was added to 2.5 mL scintillation fluid, then analyzed by a liquid scintillation counter (LS-6500, Beckman Coulter, Brea, CA). Final determination of plasma [S] was made by interpolation using a serial dilution standard curve of unlabeled S (Riechstien’s Substance S, Sigma-Aldrich, USA) ranging from 32 to 0.5 ng mL^−1^, as well as a 0 ng mL^−1^ standard, run in triplicate. All necessary precautions were taken regarding the validation of this assay: (i) the CET-M8 Ab used in our assay was validated to have high specificity for S compared to DOC, F, and A (cross-reaction < 1%); (i) lower limits of detection and quantitation of [S] were determined to be 0.25 and 0.5 ng mL^−1^, respectively; (iii) serially diluted lamprey plasma returned a linear response of calculated [S] (*m* = 1.06, *r*^2^ = 0.99); additions of 1, 2, 4, and 8 ng mL^−1^ S to lamprey plasma returned calculated [S] within 10% of expected values (*m* = 1.02).

### Radioreceptor binding assays for analysis of lamprey gill CR

Corticosteroid receptor binding assay was modified from previous methods^1,21^. Frozen gill tissue pooled from two individuals (~ 8–10 gill pouches) were placed in 300 μL of ice-cold HEPES assay buffer (25 mM HEPES. 10 mM NaCl, 1 mM monothioglycerol, pH 7.4) and homogenized on ice using a ground glass homogenizer. Homogenates were centrifuged for 10 min at 2,000*g* at 4 °C and the resulting supernatant (crude cytosolic preparation) was placed on ice for use in receptor binding assay which was carried out on ice in a 96-well plate. An aliquot of supernatant was reserved to determine protein concentration (BCA Protein Assay, Pierce). For each sample, total (B_T_) and non-specific (B_NS_) binding curves were obtained by incubating 25 μL of gill supernatant with 25 μL of assay buffer (HEPES) containing various concentrations of [^3^H]S, either alone (B_T_) or with a 500-fold excess of [cold]S (B_NS_). The final assay in each well was 50 μL and contained 4–6 mg mL^−1^ gill protein and 2.5, 5, 10, or 20 nM [^3^H]S and was incubated on ice for 2 h to allow the reaction to reach equilibrium. After incubation, 150 μL of an ice-cold dextran-coated charcoal solution (HEPES, 0.25% w/v dextran, 2.5% w/v activated charcoal) was added to each well and incubated for 10 min on ice, then centrifuged for 10 min at 2,000*g* at 4 °C to remove any unbound [^3^H]S. Finally, 100 μL of supernatant was transferred to scintillation vial containing 2 mL of scintillation fluid (ECONO-SAFE, Research Products International Corp., USA) and counted in a liquid scintillation counter (LS 6000IC, Beckman Instruments Inc., USA). Specific binding (B_S_) was calculated (B_S =_ B_T_ − B_NS_) and the corticosteroid receptor binding capacity (*B*_max_) and equilibrium dissociation constant (*K*_d_) were determined by hyperbolic regression analysis. Binding specificity was analyzed identically as above but using 1 nM [^3^H]S in competition with various concentrations (1, 10, 100, 1,000 nM) of unlabeled S, DOC, F, or A.

### Gill mRNA and protein analyses

Gill NKA and NKCC1 mRNA and protein abundance were analyzed by standard quantitative real-time polymerase chain reaction (qPCR) and Western blotting using procedures, molecular primers, and antibodies as previously validated and described in our laboratory^9^. Antibodies used were: mouse monoclonal anti-NKA α-subunit (‘α5’; RRID: AB_2166869), and mouse monoclonal anti-NKCC (‘T9’, RRID: AB_528406). Original images for Western blot analyses are provided in Supplementary Figs. S2–S5.

### Molecular phylogenetics, calculations, and statistics

Molecular phylogenetic analysis using peptide sequence data obtained from NCBI Genbank were performed using ClustalW alignment (https://www.ebi.ac.uk/clustalw) implemented by MEGA7 software^56^ using the neighbor-joining method with 1,000 replicates. The accession numbers for sequences used in our analyses were: *Homo sapiens* (GR, P04150; MR, P08235); *Mus musculus* (AR, P19091; GR, NP_001348138; MR, NP_001077375); *Gallus gallus* (GR, NP_001032915; MR, NP_001152817); *Xenopus laevis* (GR, P49844; MR, Q91573); *Danio rerio* (GR, NP_001018547; MR, NP_001093873); *Oncorhynchus mykiss* (GR, AAR87479; MR, AAS75842); *Petromyzon marinus* (CR, AAK20930). To understand the nature of the relationships between hormone or receptor and the developmentally regulated rise in gill NKA activity, we compared three theoretical curve-fitting analyses (linear, hyperbolic, and quadratic) to determine the simplest model for the relationship (most degrees of freedom) that best fit (highest *r*^2^) the data. Normality and homogeneity of variance assumptions were tested using Shapiro–Wilk and Levene’s tests, respectively. Treatment effects and comparisons between treatment groups were analyzed using Student’s t-test, one- and two-way ANOVA, and Tukey’s post hoc analyses, as indicated in figure captions. An α-value of 0.05 was selected to denote statistical significance in all analyses. All regression and statistical analyses and figures were completed using GraphPad Prism 6.0 (GraphPad Software, USA).

## Supplementary information

Supplementary Figures.
