# Supplementary material for: 11-Deoxycortisol controls hydromineral balance in the most basal osmoregulating vertebrate, sea lamprey (Petromyzon marinus)
Source: Sci Rep. 2020 Jul 22;10:12148. doi: 10.1038/s41598-020-69061-4 (PMC7376053; doi:10.1038/s41598-020-69061-4)
Supplement: Supplementary file 1 — Supplementary Figures. [file 41598_2020_69061_MOESM1_ESM.pdf]

SUPPLEMENTARY INFORMATION TO:

Title: 11-Deoxycortisol controls hydromineral balance in the most basal osmoregulating vertebrate, sea lamprey (*Petromyzon marinus*)

Authors: Ciaran A. Shaughnessy[1]*, Andre Barany[2], Stephen D. McCormick[1,3,4]

Affiliations: [1]Graduate Program in Organismic and Evolutionary Biology, University of Massachusetts, Amherst, MA, USA. [2]Departamento de Biología, Universidad de Cádiz, Cádiz, Spain. [3]Department of Biology, University of Massachusetts, Amherst, MA, USA. [4]U.S. Geological Survey, Leetown Science Center, S.O. Conte Anadromous Fish Research Center, Turners Falls, MA, USA. *To whom correspondence should be addressed (cshaughnessy@umass.edu).

[supplementary figures start on next page]

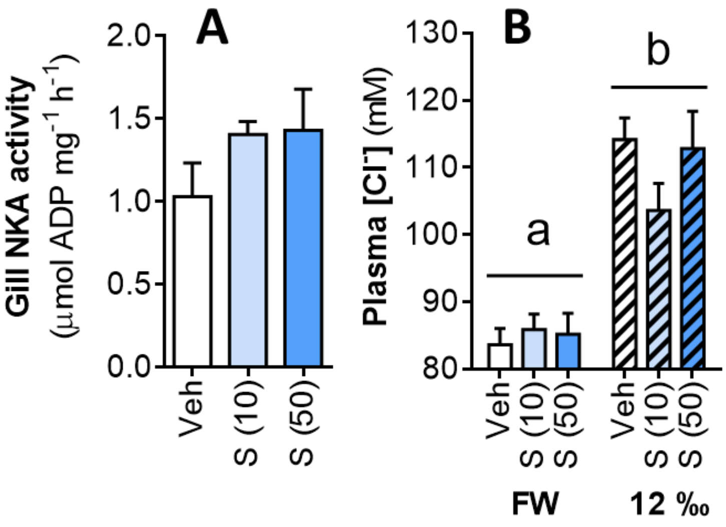

Supplementary Figure S1: Effect of 11-deoxycortisol (S) treatment in larval sea lamprey on (A) gill Na^+/K^+-ATPase (NKA) activity ($P = 0.297$; one-way ANOVA, Tukey's *post hoc*) and (B) plasma chloride concentration ($[Cl^-]$) in larval lamprey in freshwater (FW; open bars) or after exposure to seawater (SW; 12 ‰; dashed bars) 12 d after injection with an oil implant alone (Veh) or containing 11-deoxycortisol (blue bars). Dose of S is stated in parenthesis (µg g^{-1} body weight). In (B), only the effect of salinity was significant ($P < 0.001$); neither effects of S treatment ($P = 0.262$) nor the interaction ($P = 0.476$) were significant (two-way ANOVA). Values represent mean ± SEM ($n = 6$-8).

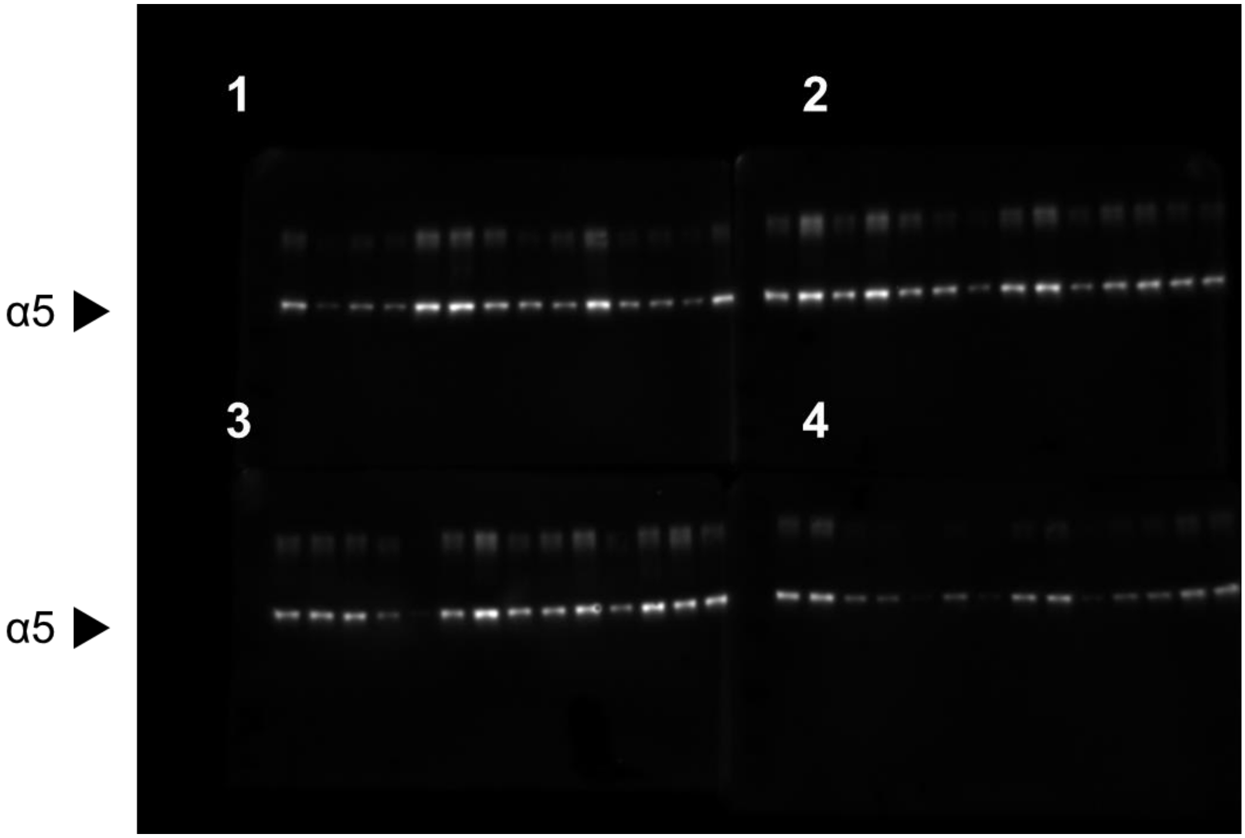

Supplementary Figure S2: Original images of Western blotting using an anti-NKA antibody (α5) to analyze gill NKA protein abundance. One image of a total of 4 membranes are shown. Values obtained from this image correspond to data in Fig. 4E.

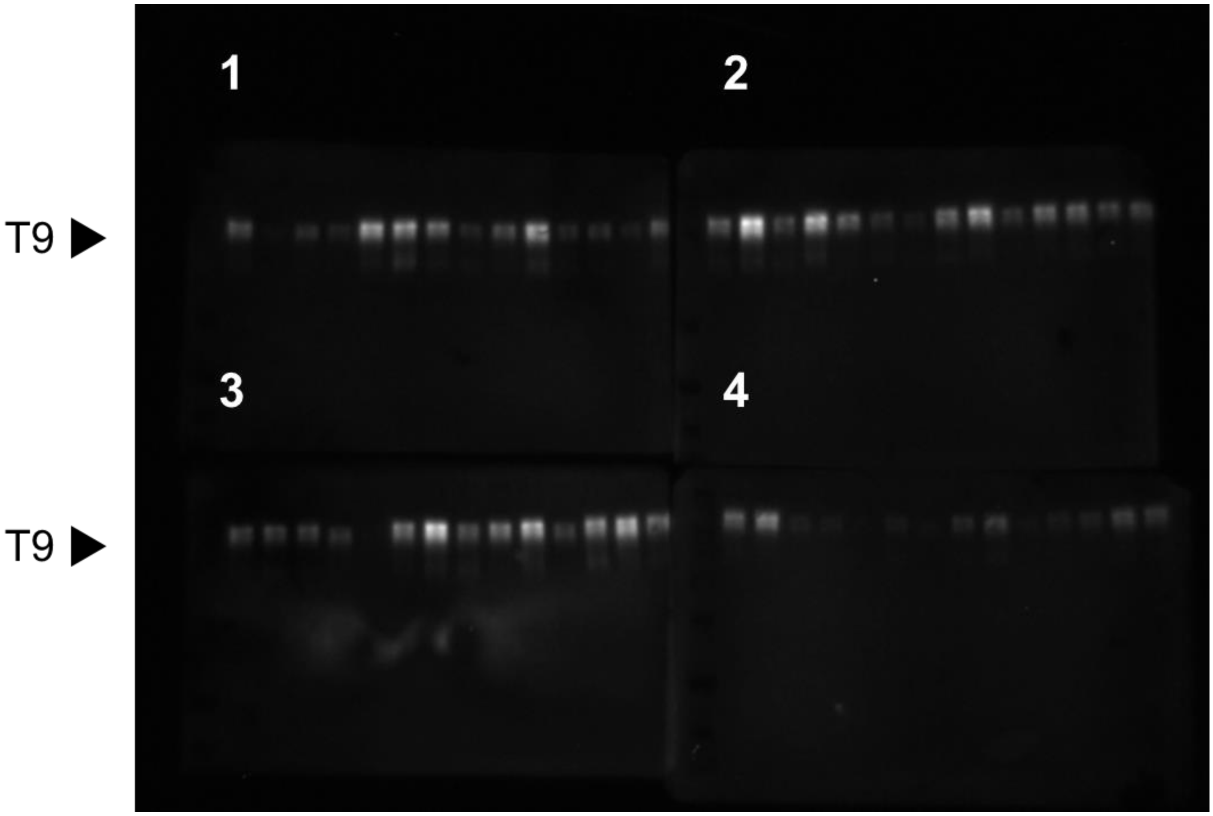

Supplementary Figure S3: Original images of Western blotting using an anti-NKCC antibody (T9) to analyze gill NKCC1 protein abundance. One image of a total of 4 membranes are shown. Values obtained from this image correspond to data in Fig. 4F.

Table of lane identification corresponding to Supplementary Figures S1 and S2:

Membrane #	Lane #	Treatment
1	2	N/A
1	3	T0
1	4	N/A
1	5	Veh
1	6	S(50)
1	7	Veh
1	8	S(10)
1	9	S(50)
1	10	Veh
1	11	S(10)
1	12	Veh
1	13	N/A
1	14	T0
1	15	N/A

Membrane #	Lane #	Treatment
2	2	N/A
2	3	Veh
2	4	T0
2	5	S(50)
2	6	S(10)
2	7	S(50)
2	8	S(10)
2	9	Veh
2	10	S(50)
2	11	T0
2	12	S(10)
2	13	N/A
2	14	T0
2	15	N/A

Membrane #	Lane #	Treatment
3	2	N/A
3	3	Veh
3	4	Veh
3	5	S(50)
3	6	T0
3	7	N/A
3	8	N/A
3	9	S(10)
3	10	S(50)
3	11	S(10)
3	12	S(50)
3	13	S(50)
3	14	N/A
3	15	N/A

Membrane #	Lane #	Treatment
4	2	N/A
4	3	S(10)
4	4	Veh
4	5	T0
4	6	S(10)
4	7	S(50)
4	8	S(10)
4	9	T0
4	10	N/A
4	11	N/A
4	12	T0
4	13	T0
4	14	N/A
4	15	N/A

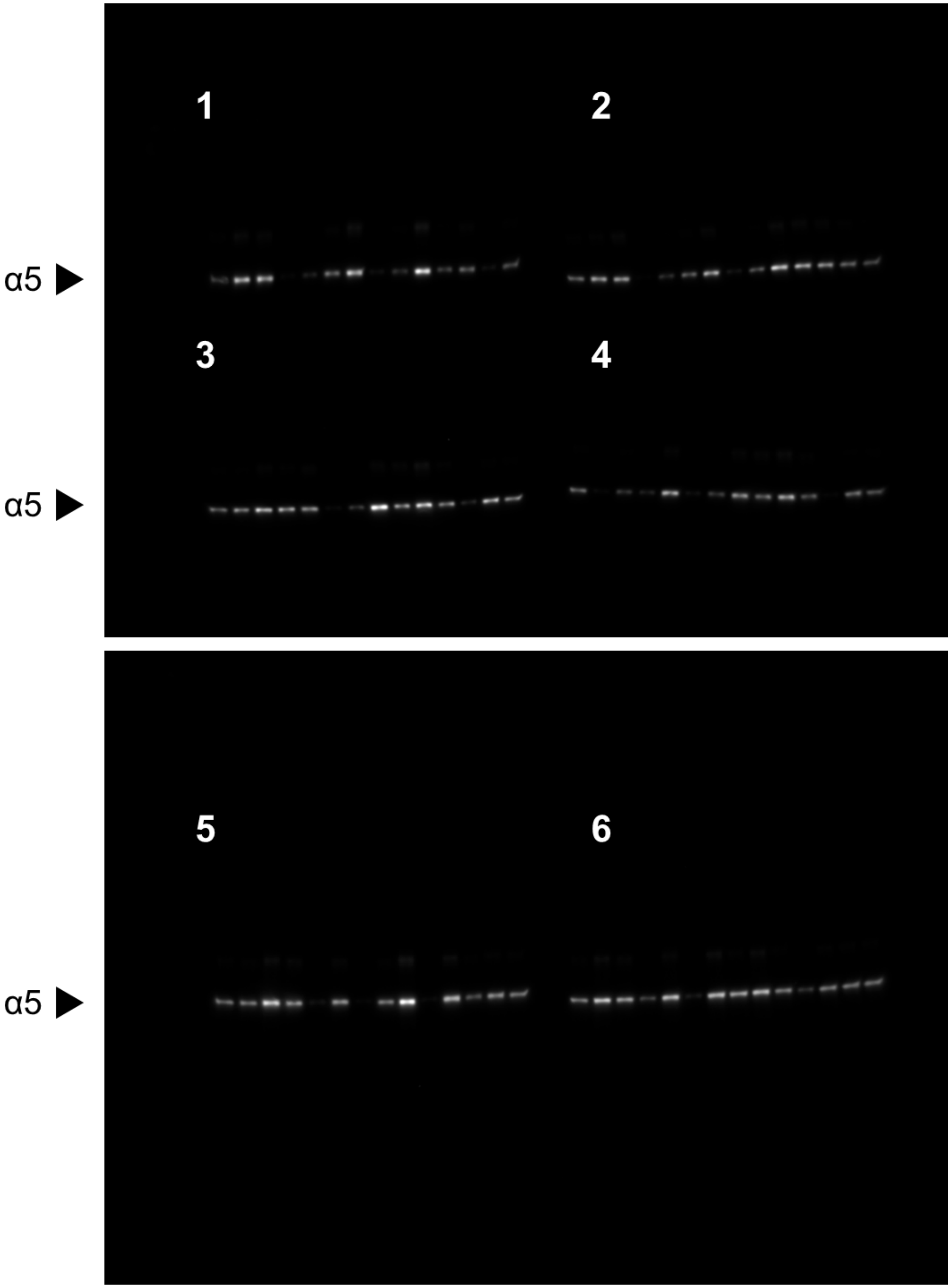

Supplementary Figure S4: Original images of Western blotting using an anti-NKA antibody (α5) to analyze gill NKA protein abundance. Two images of a total of 6 membranes are shown. Values obtained from this image correspond to data in Fig. 6C.

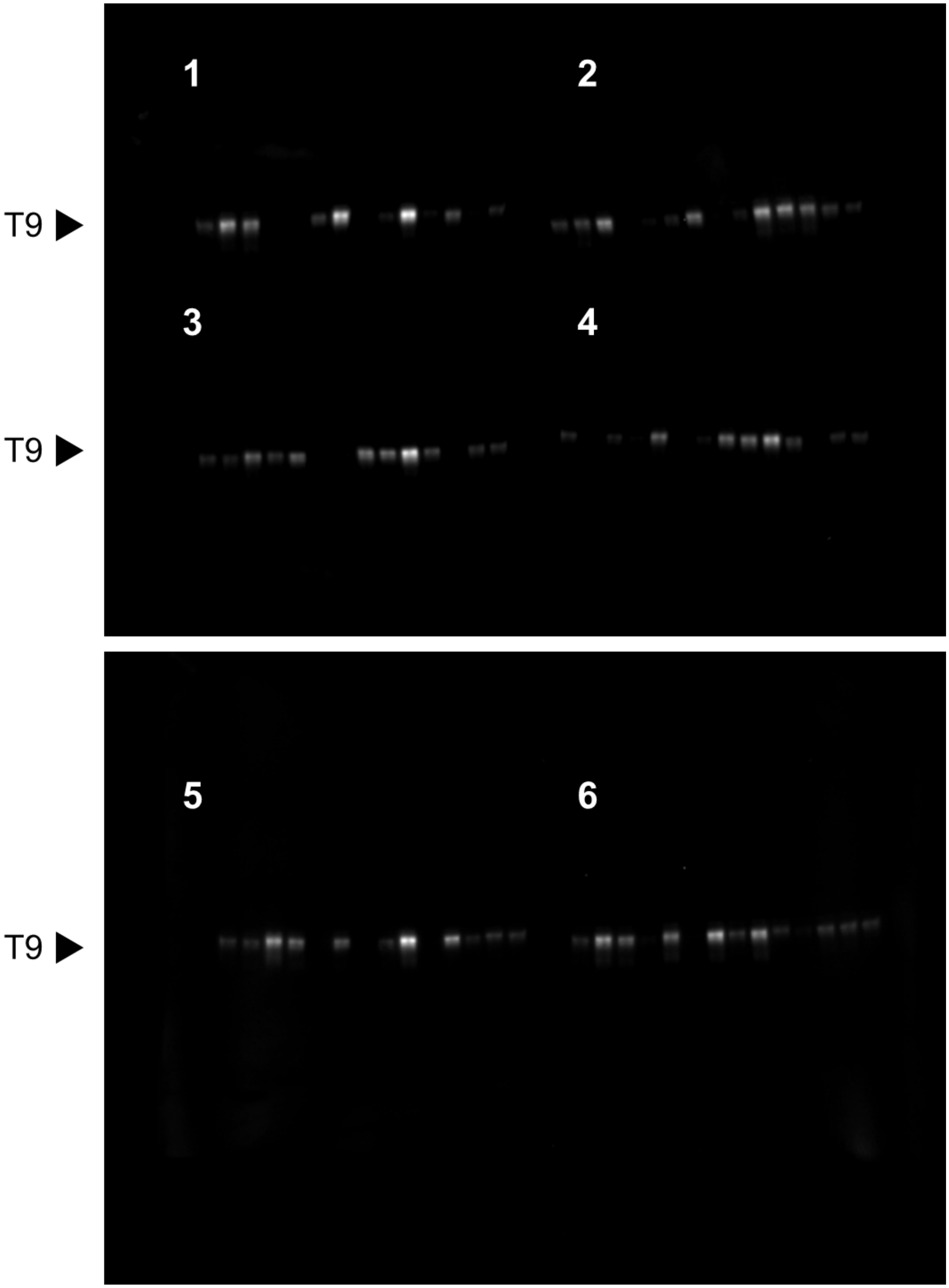

Supplementary Figure S5: Original images of Western blotting using an anti-NKCC antibody (T9) to analyze gill NKCC1 protein abundance. Two images of a total of 6 membranes are shown. Values obtained from this image correspond to data in Fig. 6D.

Table of lane identification corresponding to Supplementary Figure S4 and S5:

Membrane #	Lane #	Treatment
1	2	N/A
1	3	S(40)
1	4	DOC(10)
1	5	T0
1	6	Veh
1	7	S(5)
1	8	S(40)
1	9	DOC(10)
1	10	DOC(40)
1	11	N/A
1	12	N/A
1	13	DOC(40)
1	14	Veh
1	15	N/A

Membrane #	Lane #	Treatment
2	2	N/A
2	3	N/A
2	4	N/A
2	5	T0
2	6	Veh
2	7	S(5)
2	8	S(40)
2	9	DOC(10)
2	10	DOC(40)
2	11	N/A
2	12	N/A
2	13	S(5)
2	14	S(5)
2	15	N/A

Membrane #	Lane #	Treatment
3	2	N/A
3	3	DOC(10)
3	4	N/A
3	5	S(40)
3	6	DOC(10)
3	7	T0
3	8	DOC(10)
3	9	S(5)
3	10	N/A
3	11	N/A
3	12	DOC(10)
3	13	Veh
3	14	DOC(40)
3	15	N/A

Membrane #	Lane #	Treatment
4	2	N/A
4	3	T0
4	4	DOC(10)
4	5	Veh
4	6	N/A
4	7	T0
4	8	N/A
4	9	N/A
4	10	DOC(40)
4	11	S(40)
4	12	Veh
4	13	T0
4	14	N/A
4	15	N/A

Membrane #	Lane #	Treatment
5	2	N/A
5	3	DOC(40)
5	4	S(40)
5	5	N/A
5	6	T0
5	7	S(5)
5	8	T0
5	9	S(5)
5	10	N/A
5	11	T0
5	12	N/A
5	13	Veh
5	14	S(40)
5	15	N/A

Membrane #	Lane #	Treatment
6	2	N/A
6	3	S(40)
6	4	Veh
6	5	T0
6	6	S(40)
6	7	T0
6	8	S(5)
6	9	S(40)
6	10	DOC(40)
6	11	N/A
6	12	DOC(10)
6	13	N/A
6	14	N/A
6	15	N/A
